# Assessment of the electrochemical behaviour of Nickel-Titanium-based orthodontic wires: Effect of some natural corrosion inhibitors in comparison with fluoride

**DOI:** 10.4317/jced.55601

**Published:** 2019-05-01

**Authors:** Nassiba Fatene, Said Mansouri, Bouchra Elkhalfi, Mohamed Berrada, Khadija Mounaji, Abdelaziz Soukri

**Affiliations:** 1 Orthodontist, private practice, PhD student, Physiopathology molecular genetics and biotechnologies laboratory, Faculty of Sciences Ain Chock, Center of Health & Biotechnology, Hassan II University, Casablanca, Morocco; 2 PhD student, LIMAT-Thermostructural Materials and Polymers Team, Center of Health & Biotechnology, Faculty of science Ben M’sik, Hassan II University, Casablanca, Morocco; 3 Assistant professor, Physiopathology molecular genetics and biotechnologies laboratory, Center of Health & Biotechnology, Faculty of Sciences Ain Chock, Hassan II University, Casablanca, Morocco; 4 Professor, Ben Msik Analysis Center, Faculty of Sciences Ben Msik, Center of Health & Biotechnology, Hassan II University, Casablanca, MMorocco; 5 Professor, Physiopathology molecular genetics and biotechnologies laboratory, Faculty of Sciences Ain Chock, Center of Health & Biotechnology, Hassan II University, Casablanca, Morocco; 6 Professor, director of Physiopathology Molecular Genetics and Biotechnologies laboratory, Faculty of Sciences Ain Chock, Center of Health & Biotechnology, Hassan II University, Casablanca, Morocco

## Abstract

**Background:**

The aim of this study is to assess the corrosion resistance behaviour of Nickel-Titanium-based orthodontic wires (NiTi) in different concentrations of Sodium Fluoride (NaF) and the corrosion’s inhibitory effect of the extracts of some medicinal plants (essential oils, hydrosols and extract).

**Material and Methods:**

In this study we used NiTi (3M) and CuNiTi (ORMCO, 35°C, California) orthodontic wires. The following electrolytes were prepared: Lactate Ringer solution with additions of 0.1%, 0.5% or 1% of Sodium Fluoride and the extracts of different plants: Artemisia, Syzygium aromaticum (Clove) and Celtis australis. Corrosion resistance was studied using anodic potentiodynamic polarisation and electrochemical impedance spectroscopy measurements. At the end of the experiment, microscopic images of wires were performed. ANOVA test with the comparison of Bonferroni and Tukey tests were performed to elucidate comparisons among all groups.

**Results:**

The higher sodium fluoride concentration is related to negative corrosion potential for both NiTi and CuNiTi orthodontic wire. Hydrosols are associated to positive values of corrosion potential. CuNiTi has a lower corrosion resistance than NiTi.

**Conclusions:**

The prescription of toothpastes containing sodium fluoride should be reduced especially for patients wearing fixed orthodontic appliances. Eugenol may be considered as alternative of sodium fluoride for orthodontic patients for its anti-microbial and anti-corrosive effects.

** Key words:**Corrosion behaviour, Sodium Fluoride, Nickel-Tatanium, orthodontic wires, corrosion inhibitors, aromatical plants.

## Introduction

Wires of nickel and titanium, particularly nitinol (which contains about 50% of nickel and titanium), are the most used in orthodontics due to their interesting mechanical properties (1).

Copper added to NiTi (CuNiTi) was commercially available since the 90’s, it provides an increase in the effectiveness of tooth movement and a reduction over the orthodontic treatment’s duration (2). CuNiTi 35°C orthodontic wire is used in the DAMON system with self-ligating brackets to reduce the coefficient of attrition observed in conventional technique leading to a reduction of the number of visits and a protection of the passive layer at the surface of the wire (3).

Number of studies on the corrosion resistance of orthodontic wires have been conducted (1,4-7). Even though these wires develop a protective oxide layer on their surface, there is a metal ion release in oral cavity (8). Unfortunately, this results in toxic and allergic effects due, especially, to nickel release (9).

Furthermore, corroded orthodontic wires constitute a good habitat for oral bacteria especially for Streptococcus mutans responsible of enamel demineralisation (10).

A good understanding of the corrosion resistance behaviour of nickel-titanium based orthodontic wires is necessary to prevent ion release. Much research on the corrosive behaviour of nickel-titanium based orthodontic wires in fluoridated and acidulated medium have been conducted (5,6,11-14). However, none to our knowledge have investigated the effect of aromatic plants extracts, as alternative to sodium fluoride in toothpaste, on the corrosion properties of nickel-titanium-based orthodontic wires. It has been largely reported that fluoride helps remineralisation of enamel teeth protecting them from acid (15). Nevertheless, a negative effect of fluoride on the corrosion resistance of nickel-titanium based wires in certain experimental conditions have been described (13).

To prevent corrosion, several methods are available. Among them, there is the surface coating, the cathodic protection and the use of anticorrosive solutions and anticorrosive inhibitors. Inhibitors remain the best way to protect the surface layer of metals. They decrease or inhibit the metallic reaction with its environment, when added at low concentrations to the medium. An inhibitor can act by adsorbing on the metal surface leading to a decrease of corrosion through an increase in the anodic and/or cathodic reaction, a decrease in the speed of scattering on the metal surface, or by decreasing its electric resistance (16).

Synthetic compounds with anti-corrosive effects are very toxic for humans and for the environment (17). This leads to a special interest in the aromatic plant extracts (aqueous and oil-based extracts and essential oils) which are considered a resource of green inhibitors.

The purpose of our study is to assess the corrosion resistance behaviour of Nickel-Titanium-based orthodontic wires (NiTi) in different concentrations of Sodium Fluoride (NaF) and the corrosion’s inhibitory effect of the extracts of some plants (essential oils, hydrosols and extract) as alternatives to fluoride.

## Material and Methods

In this study, NiTi (3M) and CuNiTi (ORMCO, 35°C, California) orthodontic wires were used. Both were a 017.025-inch. For electrochemical measurements, the following electrolytes were prepared: Ringer solution (LAPROPHAN) as base of artificial saliva with additions of 0.1%, 0.5% or 1% of Sodium Fluoride (NaF; Riedel-de-Haen) and 20 μl of extracts of different plants : Artemisia, Syzygium aromaticum (Clove) and Celtis australis. Extract of Celtis australis, essential oils of Artemisia, Clove and Celtis australis and hydrosols of Clove and Artemisia were studied. Diluted HCl solution was used to adjust the pH of all solutions to 4.4. The electrochemical experiments were performed with three electrodes in a beaker tube with a saturated calomel electrode used as a reference electrode and platinum as a counter. This assembly was connected to a Potensiostat (OrigaStat - OGS080). Corrosion resistance was studied using anodic potentiodynamic polarisation at a potential scanning of 20mV/min and electrochemical impedance spectroscopy measurement at open-circuit potential, with a sinusoidal signal of 10-mV amplitude and frequencies in the range of 10kHz to 10mHz. The working electrodes were immersed in solutions for 30 minutes.

After the electrochemical experiments, microscopic photographs of wires were taken by DXR2 Raman microscope at a magnification of x50.

Statistical analysis were performed using Rstudio software. Breakdown potentials of the samples (n=3 per group) were subjected to one-way analysis of variance (*p*<0.001) among with post hoc comparison with Bonferroni and Tukey tests to elucidate multiple comparisons among different groups.

## Results

**Figure 1:**
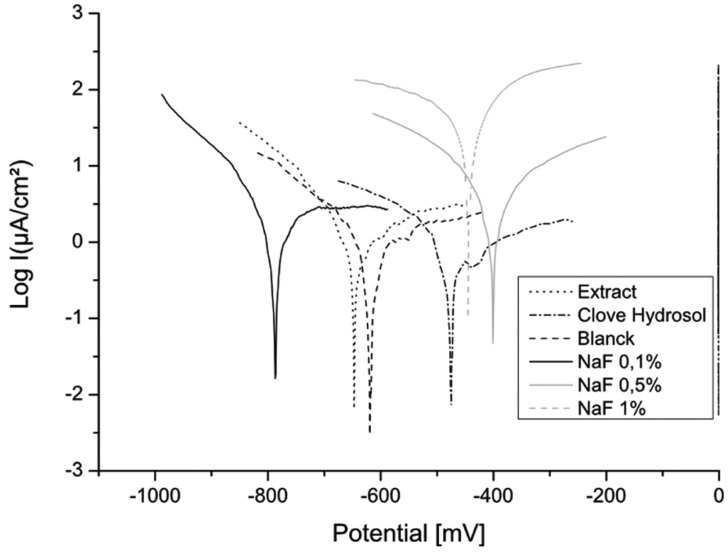
Polarisation curves of NiTi in the different concentrations of NaF.

-NiTi corrosion behaviour depending on Fluoride concentration in comparison to plant extracts:

Corrosion potential (Ecorr) measured at the end of immersion period in all solutions is presented in  Table 1 and  Table 2. Results showed that more negative Ecorr values are related to higher fluoride concentrations. The difference in Ecorr obtained in the lowest F- concentration (0.1%) and the highest F- concentration (1%) was -1102mV. Figure 1 shows polarisation curves in different fluoride concentrations.

**Table 1:**
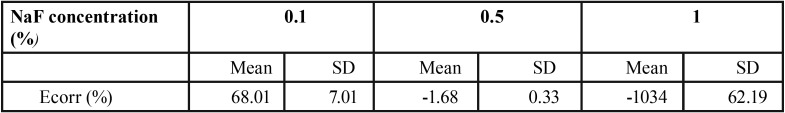
Mean and standard deviation variation of the Potential (Ecorr) of NiTi depending on NaF concentrations.

**Table 2:**
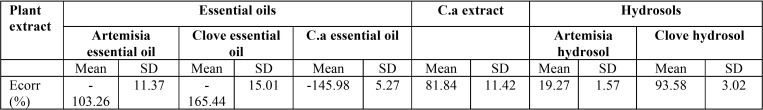
Mean and standard deviation variation of the Potential (Ecorr) of NiTi depending on plant extracts.

In solution containing 0.1% NaF the material remains passive until its breakdown potential, beyond which a breakdown of the passive layer occurs. From 0.5% fluoride ion concentration and above, the current densities are higher, which suggests active dissolution of the material.

**Figure 2:**
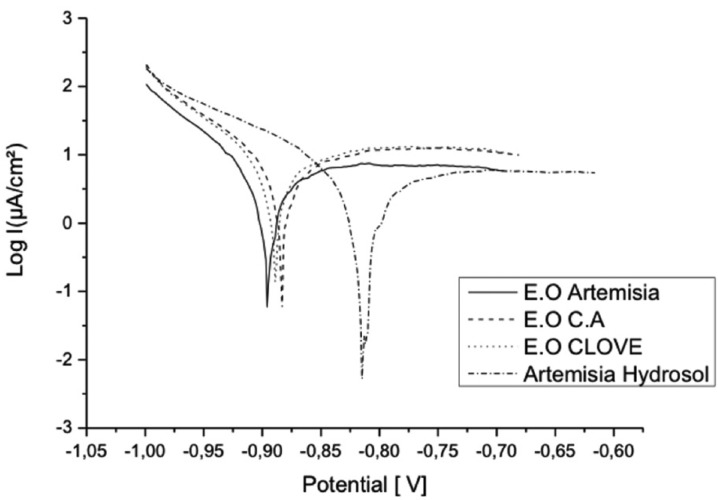
Polarisation curves of NiTi in the different plant extracts solutions.

In solutions containing extracts of plants, hydrosols (Clove and Artemisia) act like anodic inhibitors of corrosion (Figs. 1,2). While the effect of the extract and essential oils is minor.

**Figure 3:**
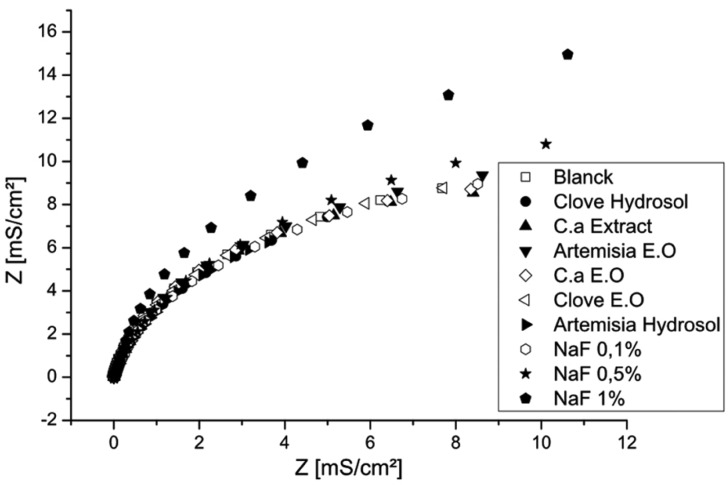
Electrochemical impedance spectroscopy of NiTi in different media.

Electrochemical impedance spectroscopy (EIS) measurements (Fig. 3) shows capacitive arcs in all fluoride concentrations. The diagrams reveal that the polarization resistance (Rp) decreases with an increase in fluoride concentration. This justifies that the decrease in corrosion resistance of NiTi is related to higher fluoride concentrations.

The analysis of variance shows that there is a significant difference between groups (*p*<0.001) and the correction of Bonferroni indicated that there is a significant difference in the potential of corrosion of NiTi wire between alomost the three concentrations of NaF and the other extracts, between essential oils and hydrosols and between the two hydrosols. These results were confirmed by the Tukey test.

-CuNiTi corrosion behaviour depending on fluoride concentration in comparison to plant extracts.

Ecorr measured at the end of immersion period in all solutions and presented in table III shows that negative values are related to solutions containing all concentrations of sodium fluoride and this containing essential oils. Positive values of Ecorr are observed in solutions containing hydrosols and oil-based extract ( Table 3).

**Table 3:**
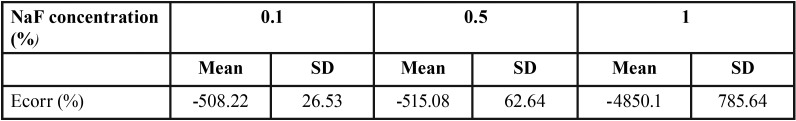
Mean an standard deviation variation of Potential (Ecorr) of CuNiTi depending on Fluoride.

**Figure 4:**
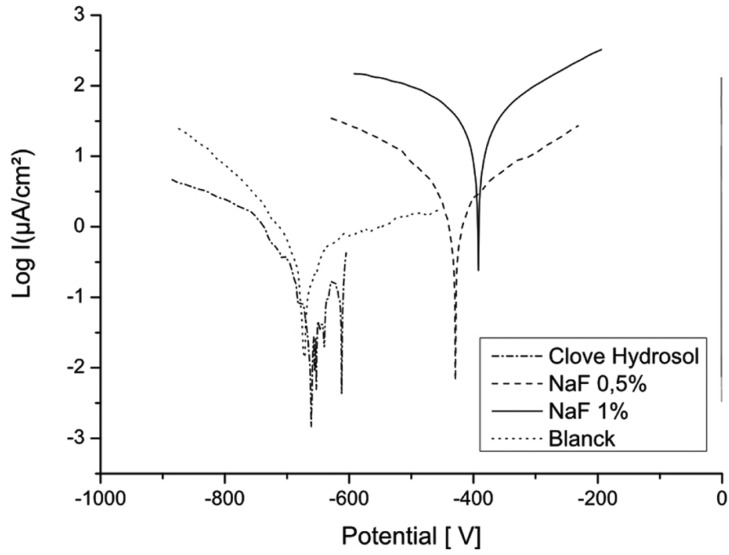
Polarisation curves of CuNiTi in the different concentrations of NaF.

**Figure 5:**
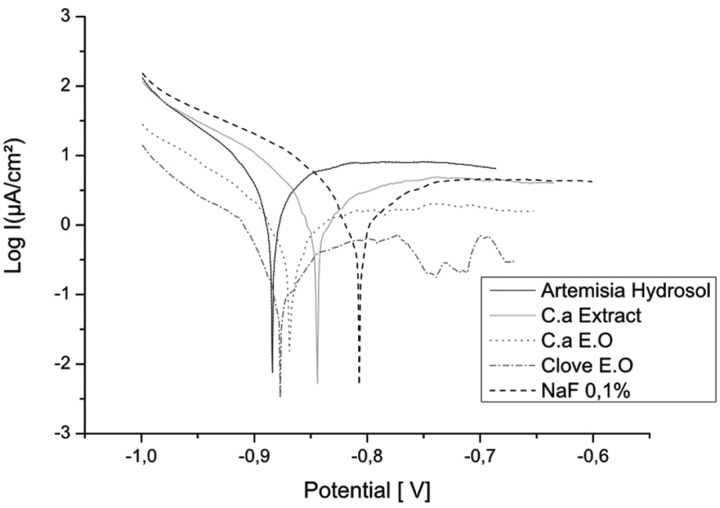
Polarisation curves of CuNiTi in the different plant extract solutions.

**Figure 6:**
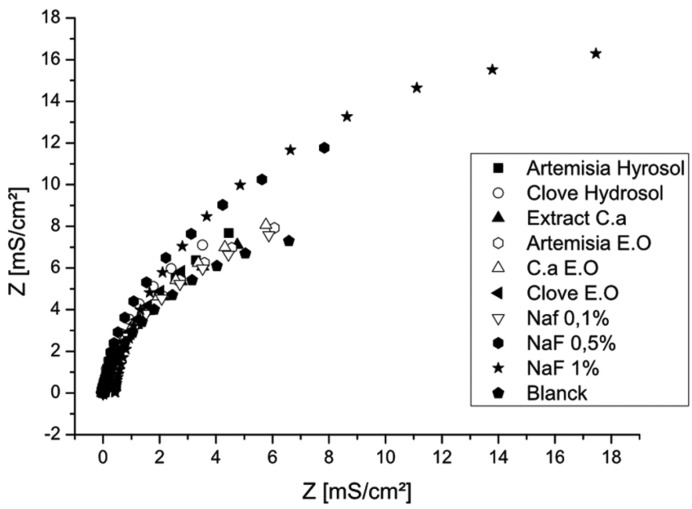
Electrochemical impedance spectroscopy of CuNiTi in different media.

Figures 4 and 5 show the polarisation curves in different solutions and confirm that Clove and Artemisia hydrosols act like inhibitors of corrosion. These results are confirmed with the electrochemical impedance spectroscopy measurements (Fig. 6). The EIS graphic confirms lower impedance of the passivation film when fluoride is present in solutions.

For CuNiTi, variance analysis shows that there is a significant difference between groups (*p*<0.001) and the correction of Bonferroni indicated that there is a significant difference only between the higher concentration of NaF (1%) and all other groups. This was confirmed by the Tukey test.

-Comparative analysis Between NiTi and CuNiTi Through Electrochemical Measurements

A comparison in the corrosion behaviour between NiTi and CuNiTi was carried out. CuNiTi presented lower Ecorr than does NiTi in solutions containing Fluoride. In general CuNiTi have a lower corrosion resistance in fluoridated and acidulated medium than NiTi.

-Microscopic analysis 

The analysis of microscopic photographs of NiTi and CuNiTi after immersion time shows that in low concentrations of NaF, we noted localised pitting and coloration all over the wires. By increasing the concentration of NaF (1% and above), we observe general and large pitting in all the surface of the wire.

**Figure 7:**
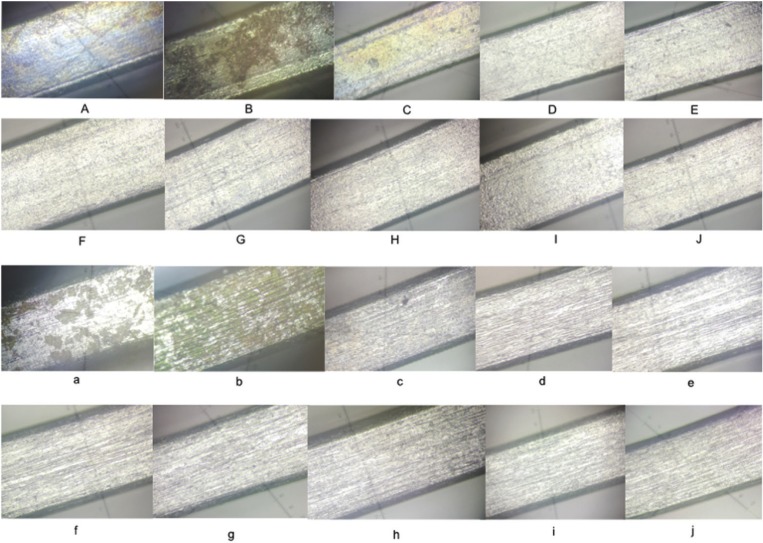
A-J: NiTi wire, a-j: CuNiTi wire. A,a: 0.1% NaF; B,b: 0.5% NaF; C,c: 1% NaF; D,d: As received wire; E,e: Artificial saliva ; F,f: Clove hydrosol; G,g: C.a essential oil ; H,h:Clove essential oil ;I,i: Artemisia hydrosol; J,j: C.a extract.

For both wires, in solutions containing Clove hydrosol there is no pitting and the surface remains uncoroded (Fig. 7), ( Table 4).

**Table 4:**
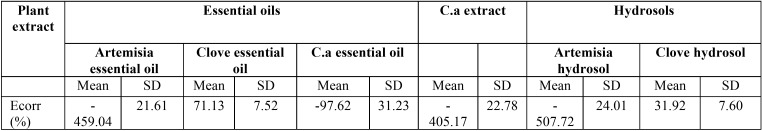
Mean and standrad deviation variation of Potential (Ecorr) of CuNiTi depending on plant extracts.

## Discussion

Our results suggest that higher fluoride concentrations lead to a decrease of corrosion resistance of NiTi. Similar studies in the literature reported the same conclusions (1,5,6). Previous investigations demonstrated that by increasing the concentration of fluoride, the passive layer is lost (14). This would indicate that fluoride is aggressive for NiTi alloys. This finding is confirmed by SEM analysis of the surface roughness of NiTi wires after immersion in fluoride solutions which suggested that in low concentrations, localised pitting are observed and in higher concentrations the corrosion become generalised as we noted in our study (13). Eliades in his review reported that NiTi may present crevices and pores that represent sites susceptible to corrosion (18).

We observed that the corrosion behaviour of CuNiTi is different from that of NiTi. In low concentrations, we noticed that the Ecorr is high. By increasing the concentration of fluoride, the corrosion resistance of CuNiTi decreases and the passivation layer is broken. This could be explained by the irregularities present in the surface of as received CuNiTi (2). Comparing the corrosion resistance of CuNiTi and NiTi, we can say that CuNiTi had a less corrosion resistance than NiTi in all solutions.

The choice of this specific plants used in this study was based on previous publications. After a literature review, we founded that some plants are used as green inhibitors of corrosion of metals in industry (19-21). To our knowledge, no study has been carried out on orthodontic alloys. The choice of Artemesia and Celtis australis was based on previous studies that demonstrated an anticorrosive effect on other metals (22,23). We also chose to test the anticorrosive effect of Clove (Syzygium aromaticum) essential oil and hydrosol for their antibacterial properties against oral bacteria and its widespread use in dental practice (24).

In this study, we found that hydrosols have a more positive effect on NiTi’s corrosion resistance than have the essential oils and the extract of Celtis australis. Hydrosol or aromatic water is the aqueous phase obtained by distillation of herbs in addition to the essential oil (25). A study conducted on persian herbs demonstrated that the chemical composition of essential oils is very different from their aromatic water (26). We observed the best corrosion resistance of NiTi wires in the solution containing clove hydrosol.

In order to understand our finding, a literature research about the composition of clove essential oil was carried out. Eugenol is actually the major constituent of clove essential oil, it is a hydroxyphenyl propene that demonstrated a great biological activity with an antimicrobial action on oral pathogens (27,28). These results suggest that Eugenol could be used in toothpastes and mouthwashes especially for patients with fixed orthodontic appliances instead of Sodium Fluoride.

Further investigations, especially the characterisation by GC-MS has to be done in order to know exactly the difference of the composition between hydrosols and essential oils used in our study. This constitutes the next step of our work to understand why hydrosols act like inhibitors of corrosion rather than the corresponding essential oils and to determine the minimal inhibitory concentration of eugenol.

## Conclusions

This study reported that.

- Increased Sodium Fluoride concentrations lead to decreased corrosion resistance of Nickel-Titanium-based wires.

- CuNiTi had a less corrosion resistance than NiTi in fluoridated and acidulated solutions.

- Clove hydrosol which contains mainly Eugenol, shows the highest corrosion resistance of both NiTi and CuNiTi wires.

- The prescription of toothpastes containing sodium fluoride should be reduced especially for patients wearing fixed orthodontic appliances.

- Eugenol may be considered as alternative of sodium fluoride for orthodontic patients for its anti-corrosive and anti-microbial effects.

- Further investigation have to be done to confirm this results and to determine the minimal inhibitory concentration of Eugenol on NiTi-based orthodontic wires.
